# Prevalence, Trends, and Incidence of Alcohol Withdrawal Symptoms: Analysis of General Population and Clinical Samples

**Published:** 1998

**Authors:** Raul Caetano, Catherine L. Clark, Thomas K. Greenfield

**Affiliations:** Raul Caetano, M.D., Ph.D., and Thomas K. Greenfield, Ph.D., are senior scientists and Catherine L. Clark, Ph.D., is an associate scientist at the Alcohol Research Group, Public Health Institute, Berkeley, California

**Keywords:** AOD withdrawal syndrome, symptom, prevalence, incidence, trend, AOD consumption, addiction care, drinking and driving, offender, treatment, racial differences, ethnic differences, white American, African American, Hispanic, demographic characteristics, survey, clinical study, literature review

## Abstract

Analyses of the prevalence and incidence of withdrawal symptoms in the general population can provide an estimate of the frequency of alcohol dependence in the population. Similar analyses in people who are being treated for alcoholism or alcohol-related problems can identify the need for and specific types of treatment required for these populations. Three national surveys found that the prevalence of withdrawal symptoms was relatively low in the general population and has remained stable over the past 15 years. The likelihood of experiencing withdrawal symptoms increased with increasing alcohol consumption. No differences in the prevalence of withdrawal symptoms existed among ethnic groups in the general population. In a sample of patients undergoing alcoholism treatment, the prevalence of withdrawal symptoms generally was high, with lower rates among blacks than among whites and Hispanics. The prevalence of withdrawal symptoms in people undergoing treatment after being convicted of driving under the influence fell between that of the general population and that of the treatment sample.

The presence of alcohol withdrawal symptoms is an important indicator of serious addiction to alcohol and serves as one of the diagnostic criteria for alcohol dependence according to the World Health Organization’s *International Classification of Diseases* and the American Psychiatric Association’s *Diagnostic and Statistical Manual of Mental Disorders* (DSM). (For more information on the definition and significance of alcohol withdrawal, see the article by Saitz, pp. 5–12.) This Epidemiologic Bulletin summarizes current estimates of the prevalence, trends, and incidence of withdrawal symptoms in both general population and clinical samples in the United States. (For more detailed information about the research reviewed in this article, see Caetano and Clark 1998; Caetano and Kaskutas 1996; Caetano and Schafer 1996.) Analyses of withdrawal symptomatology in the general population provide a rough indicator of the extent to which alcohol dependence problems are present in our society. Analyses of withdrawal symptomatology in clinical samples can help identify groups of drinkers who may need treatment as well as the types of services required to adequately meet the needs of specific groups, particularly ethnic minorities. The comparison of withdrawal symptom rates in general population and clinical samples allows evaluation of the overall significance of the phenomenon.

## Methods

### General Population Sample

The general population data presented here were derived from the 1984, 1990, and 1995 National Alcohol Surveys. These cross-sectional^1^ surveys are part of a series of nationwide surveys conducted by the Alcohol Research Group to examine trends in alcohol consumption patterns and alcohol-related problems throughout the United States. Fieldwork for these surveys was conducted by the Temple University Institute of Survey Research. To obtain samples that were generalizable to the entire U.S. population, a complicated sampling design was developed. The sample for each survey was derived from 100 primary sampling units located throughout the 48 contiguous States. These units represent specific geographic areas selected according to their location (e.g., region of the country) and population size (Babbie 1973). For each survey, subjects were randomly selected, using a multistage area probability procedure, from among all people age 18 and older living in households in the sampling units. Both the 1984 and 1995 surveys oversampled blacks and Hispanics to allow researchers to analyze withdrawal symptoms among these ethnic groups with greater statistical precision.

In the 1984 survey, 5,177 respondents were interviewed, with a response rate of 74 percent of all people contacted. In the 1990 survey, 2,058 respondents were interviewed, and the response rate was 71 percent. The 1995 survey of 4,803 respondents had a response rate of 77 percent. In all three surveys, trained interviewers collected the data in face-to-face interviews that lasted, on average, 1 hour. The interviews were conducted in the respondents’ homes using standardized questionnaires and, as appropriate, involved bilingual interviewers and instruments translated into Spanish.

### Longitudinal Survey of 1984–1992

The 1984 National Alcohol Survey described previously provided the baseline data for a longitudinal study in which a subsample of the respondents were reinterviewed in 1992. Followup interviews were successfully completed with 2,214 respondents, resulting in an overall response rate for the followup of 70 percent. To determine whether nonrespondents in the followup represented a specific subgroup of the original respondents (which could bias the results), the 1984 data were used to compare the respondents who were reinterviewed with those who were not. The results of these comparisons did not reveal any significant differences among the ethnic groups in the distribution of drinking patterns, mean number of drinks consumed per month, or number of alcohol problems present in the past 12 months.

### Clinical Samples

#### Treatment Sample

The treatment sample consisted of 256 white, 263 black, and 212 Mexican-American men admitted to five public detoxification and residential alcoholism treatment programs. Four of these programs were located in the city of San Jose in Santa Clara County, California; the fifth treatment program was located in San Mateo County, California. Interviews were conducted by trained interviewers in the program facilities between June 1993 and May 1994 using standardized questionnaires. The response rates were 91 percent for whites, 88 percent for blacks, and 90 percent for Mexican-Americans.

#### DUI Sample

This sample included 248 whites and 252 Mexican-Americans interviewed at five treatment programs for persons convicted of driving under the influence (DUI) in the city of San Jose. These programs were educational in nature and were mandated for people convicted for DUI. The treatment programs accepted clients who had been driving under the influence of either alcohol or other drugs; however, most program participants (and people in the study) had been convicted solely for driving under the influence of alcohol. Interviews were conducted by trained interviewers in the program facilities between February and September of 1997 using standardized questionnaires. The response rates were 71 percent for whites and 84 percent for Mexican-Americans.

Because approximately 40 percent of the respondents in both clinical samples were dependent on drugs other than alcohol, the interviewers were trained to distinguish specifically between symptoms of withdrawal from alcohol and symptoms of withdrawal from other drugs. To this end, the interviewers posed clearly identifying questions about alcohol withdrawal and probed for specific answers when necessary.

### Operational Definitions

In the national surveys of 1984, 1990, 1992, and 1995, respondents were asked to report whether they had experienced any of the following withdrawal symptoms in the 12 months before the interview:

- Drank in the morning to “get over” the effects of last night’s drinking
- Experienced shaking hands the morning after drinking
- Awakened sweating because of drinking
- Needed a drink to keep from shaking
- Became sick or vomited after drinking
- Felt depressed, irritable, and/or nervous after drinking.

In both the treatment and DUI samples, information about withdrawal symptoms was collected using the fourth edition of DSM’s Composite Diagnostic Interview Schedule (CIDI–SAM) (Keating et al. 1992).

#### Ethnic Identification

The main identifier of ethnic status was the ethnicity of the family of origin. Respondents were asked, “Which of these groups describes your family of origin?” Four categories were provided for self-identification, including (1) black of Hispanic origin (Latino, Mexican, Central or South American, or any other Hispanic origin); (2) black, not of Hispanic origin; (3) white of Hispanic origin (Latino, Mexican, Central or South American, or any other Hispanic origin); and (4) white, not of Hispanic origin. Respondents who selected either “black of Hispanic origin” or “white of Hispanic origin” were classified as Hispanic. Respondents who selected the category “black, not of Hispanic origin” were classified as black. Subjects who said that their family of origin was “white, not of Hispanic origin” were classified as white.

## Results

### Characteristics of the Samples

The demographic characteristics of the respondents varied somewhat among the five samples included in this project (see table 1). For example, the mean ages of the general population samples were slightly older than those of the treatment and DUI samples. Both the general population and DUI samples had higher percentages than the treatment sample of individuals with an annual family income above $20,000. In addition, the percentages of individuals who had completed high school and who were married were higher and the percentage of respondents who were divorced was lower among the general population samples compared with the treatment and DUI samples. As expected, the mean number of alcoholic drinks consumed per week was considerably higher in the treatment sample than in the general population samples. The mean alcohol consumption in the DUI sample fell between that of the general population samples and the treatment sample.

### Trends in Withdrawal Symptom Prevalence in the U.S. General Population

Analyses of the responses from the three general population samples demonstrated that alcohol withdrawal symptoms generally were rare among the U.S. general population. Most of those symptoms were reported by fewer than 5 percent of the respondents (see table 2). The two most prevalent symptoms, both for men and women, were (1) becoming sick or vomiting after drinking and (2) feeling depressed, irritable, or nervous after drinking. As a result of the wording of the questions, however, it is possible that heavy drinkers who responded affirmatively to these items were experiencing the effects of heavy-drinking episodes rather than withdrawal symptoms. No significant differences in withdrawal symptoms existed across the surveys, indicating that the prevalence of these symptoms was stable over the survey years.

The 1995 survey also assessed the prevalence of two additional, severe withdrawal symptoms. These symptoms included seeing, feeling, or hearing things that were not really there (i.e., hallucinations) and having fits or seizures (i.e., convulsions) when the effects of drinking were wearing off. The prevalence of both symptoms among the general population was low. Among male respondents, 1 percent each of whites, blacks, and Hispanics reported experiencing hallucinations; no one reported experiencing convulsions. None of the female respondents reported having either hallucinations or convulsions.

### Relationship Between Alcohol Consumption and Presence of Withdrawal Symptoms

A series of multiple logistic regression analyses was conducted to examine the association between the level of alcohol consumption and the presence of withdrawal symptoms while controlling for numerous sociodemographic characteristics. Multiple logistic regression is a statistical technique that allows one to find the best fitting model to describe the relationship between an outcome variable and a set of explanatory variables (Hosmer and Lemeshow 1989; Tabachnick and Fidell 1989). In these analyses, the respondents’ reports of one or more withdrawal symptoms served as the outcome variable. The set of explanatory variables included alcohol consumption (i.e., the average number of drinks consumed per week in the past 12 months), age, gender, income, education, marital status, employment, ethnicity, and the importance of religion in the respondent’s life. These particular sociodemographic variables were chosen because they had previously been shown to be predictive of alcohol consumption patterns. Because multistage sampling designs were used in the four general population surveys, standard errors were adjusted using the Software for Survey Data Analysis (SUDAAN) statistical package.

The data from the 1984 survey demonstrated that an increase in a person’s alcohol consumption of 10 drinks per week increased by approximately twofold the likelihood of that person reporting a withdrawal symptom (odds ratio = 2.01, 95-percent confidence interval = 1.65–2.45). The data from the 1990 and 1995 surveys generated similar results (1990: odds ratio = 2.71, 95-percent confidence interval = 2.22–3.32; 1995: odds ratio = 2.22, 95-percent confidence interval = 1.81–2.71).

### Trends in Withdrawal Symptom Prevalence Among Ethnic Subgroups

Using data from the 1984 and 1995 general population surveys, both of which had oversampled blacks and Hispanics, the prevalence of withdrawal symptoms also was assessed separately for men and women of the three ethnic subgroups. These analyses revealed that the prevalence of all withdrawal symptoms was low (i.e., between 0 and 8 percent) and did not differ significantly among whites, blacks, and Hispanics (see table 3). Moreover, no significant differences existed between the 1984 and 1995 prevalence rates of withdrawal symptoms in the groups, indicating a stable trend.

### Incidence of Withdrawal Symptoms Between 1984 and 1992

Data from the 1984–1992 longitudinal survey were used to determine the incidence of withdrawal symptoms in the general population. The incidence of a withdrawal symptom was defined as the percentage of drinkers who reported the withdrawal symptom in 1992 out of all drinkers who had not reported that withdrawal symptom in 1984. Among male respondents, the percentage of subjects who reported having developed one or more withdrawal symptoms was higher among blacks and Hispanics than among whites (see table 4). Similarly, the incidence of drinking in the morning to “get over” last night’s drinking was higher among black and Hispanic men than among white men. Among women, incidence rates for all withdrawal symptoms analyzed were low and did not differ significantly among the ethnic groups.

### Prevalence of Withdrawal Symptoms in the Treatment Sample

All withdrawal symptoms analyzed had high prevalence rates in the treatment sample, regardless of the respondents’ ethnicity (see table 5). The prevalence rates for individual symptoms ranged from 8 to 86 percent. Moreover, prevalence rates were generally lower among blacks than among whites and Hispanics. Few respondents experienced the most severe indicators of withdrawal, including delirium tremens, seizures, or hallucinations.

### Prevalence of Withdrawal Symptoms in the DUI Sample

In general, the prevalence of withdrawal symptoms in the DUI sample fell between those observed in the general population samples and in the treatment sample. Moreover, for almost all withdrawal symptoms assessed, the prevalence rates were higher among whites than among Mexican-Americans undergoing treatment after a DUI conviction (see table 6). The most prevalent indicators of withdrawal were those that are less severe, such as inability to sleep (i.e., insomnia), anxiety, depression, and headaches. These symptoms were reported by approximately one- to two-thirds of the respondents, regardless of ethnicity. Drinking to relieve withdrawal symptoms was reported by about one-fifth of the whites and one-tenth of the Mexican-Americans.

## Discussion

The results presented here can help researchers gauge the distribution of withdrawal symptoms in clinical populations and in the U.S. general population. The rates of withdrawal symptoms were found to be much higher in the clinical samples than in the general population samples. Furthermore, differences in the prevalence of withdrawal symptoms also distinguished the alcoholism treatment sample from the DUI sample.

The results also indicate that the levels of withdrawal symptoms in the U.S. general population have remained stable over the past 15 years. This finding is interesting, because per capita consumption has decreased from 2.76 gallons of absolute alcohol in 1981 to 2.21 gallons in 1994 (Williams et al. 1996). Thus, the decrease in the overall alcohol consumption rate has not led to a corresponding reduction in the prevalence of withdrawal symptoms. Several possible explanations exist for this observation. For example, the declining consumption could be more concentrated among lighter drinkers who experience no withdrawal symptoms. Alternatively, the decline in consumption may not have been strong enough to trigger parallel declines in the prevalence of alcohol-related problems, including withdrawal symptoms. This possibility is supported by other survey data suggesting that other alcohol-related problems (e.g., health-related, work-related, and financial problems) also have not declined in the U.S. population over the past decade (Caetano and Clark 1998; Midanik and Clark 1995).

### Differences Among Ethnic Groups

The results of the analyses presented in this article also show substantial differences among the ethnic groups studied in the prevalence and incidence of withdrawal symptoms. For example, in the treatment sample, the prevalence of withdrawal symptoms was highest among white men, followed by Hispanic men and black men. This finding corresponds with other analyses of the same set of data, which demonstrated that alcohol dependence was more prevalent among white and Hispanic respondents than among black respondents (Caetano and Schafer 1996). Furthermore, black clients in this sample were more likely to be cocaine dependent than alcohol dependent and were therefore more likely to experience cocaine withdrawal symptoms rather than alcohol withdrawal symptoms.

### Limitations

The findings of these analyses are subject to two major limitations. First, the data are based solely on self-reports of the study participants. Ideally, this information should be confirmed through additional reports on a respondent’s withdrawal symptomatology from his or her friends, family members, co-workers, or health care professionals. Second, the comparison of nationwide general population data with clinical data from two counties in Northern California may be somewhat misleading. Certainly, rates of withdrawal symptoms in California treatment settings may not correspond to rates of withdrawal symptoms in treatment settings in other States. Consequently, further analyses using samples representing treatment settings across the United States would be helpful.

## Conclusions

Withdrawal symptoms occur with a relatively low frequency among the U.S. general population, regardless of the ethnicity of the sample studied. Moreover, the prevalence of withdrawal symptoms has remained stable between 1984 and 1995. Conversely, withdrawal symptoms are a relatively common occurrence in people undergoing alcoholism treatment and among clients in DUI treatment programs. Careful assessment of the severity of withdrawal symptomatology among these individuals will help clinicians to gauge the severity of their alcohol intake, the severity of their dependence on alcohol, and the type and intensity of treatment necessary and appropriate.

## Figures and Tables

**Table 1:** Demographic Characteristics of the Respondents From the Five Samples

	Dataset				
	U.S. General Population			Clinical Population	
	
Characteristic	1984^a^	1990^b^	1995^c^	Treatment Sample^d^	DUI^e^
Mean age (years) Percentage with household	43.0	46.6	44.5	36.1	34.5
income over $20,000	53.9	63.4	62.8	26.2	64.2
Percentage of high school graduates	74.7	75.6	80.5	47.5	63.8
Percentage married	64.7	66.0	65.2	17.5	39.4
Percentage separated/divorced	9.3	11.2	11.8	38.6	20.3
Mean number of drinks consumed weekly	5.9	3.7	4.4	62.1	21.5

a Respondents to the 1984 General Population National Alcohol Survey.

b Respondents to the 1990 General Population National Alcohol Survey.

c Respondents to the 1995 General Population National Alcohol Survey.

d Respondents recruited from five alcoholism treatment programs in Northern California.

e Respondents recruited from five treatment programs for people convicted of driving under the influence (DUI) in San Jose, California.

**Table 2:** Prevalence of Withdrawal Symptoms in Three National Alcohol Surveys by Gender

	Men (%)			Women (%)		
	
	1984 (*n* = 2,093)	1990 (*n* = 869)	1995 (*n* = 2,220)	1984 (*n* = 3,128)	1990 (*n* = 1,189)	1995 (*n* = 2,705)
Drank in the morning to get over last night’s drinking	2	2	3	0	0	1
Experienced shaking hands morning after drinking	3	3	3	2	1	1
Awakened sweating because of drinking	2	4	2	1	2	1
Needed a drink to keep from shaking or getting sick	NA	2	1	NA	0	0
Became sick or vomited after drinking	NA	12	8	NA	8	5
Was depressed, irritable, or nervous after drinking	NA	7	6	NA	5	4

NOTE: NA = not available because item was not asked in some surveys.

**Table 3:** Prevalence of Withdrawal Symptoms in Two National Alcohol Surveys Oversampling Blacks and Hispanics

	Men (%)						Women (%)					
	White		Black		Hispanic		White		Black		Hispanic	
	1984 (*n* = 743)	1995 (*n* = 754)	1984 (*n* = 719)	1995 (*n* = 644)	1984 (*n* = 599)	1995 (*n* = 764)	1984 (*n* = 1,034)	1995 (*n* = 883)	1984 (*n* = 1,222)	1995 (*n* = 936)	1984 (*n* = 831)	1995 (*n* = 817)
Drank in the morning to get over last night’s drinking	1	3	7	4	2	4	0	0	1	1	0	1
Experienced shaking hands morning after drinking	3	3	5	3	2	2	2	1	1	1	0	2
Awakened sweating because of drinking	2	2	2	3	1	2	1	1	1	1	0	1
Became sick or vomited after drinking	NA	8	NA	7	NA	8	NA	5	NA	3	NA	4
Was depressed, irritable, or nervous after drinking	NA	7	NA	5	NA	4	NA	4	NA	3	NA	2

NOTE: NA = not available because item was not asked in some surveys.

**Table 4:** Incidence of Withdrawal Symptoms in a National Longitudinal Sample––1984 and 1992 (in Percent^a^)

	Men (%)			Women (%)		
		
	White	Black	Hispanic	White	Black	Hispanic
Drank in the morning to get over last night’s drinking	**1** (*n* = 360)	**4** (*n* = 296)	**8** (*n* = 312)	**0** (*n* = 419)	**1** (*n* = 401)	**1** (*n* = 360)
Experienced shaking hands morning after drinking	**1** (*n* = 352)	**2** (*n* = 310)	**2** (*n* = 329)	**1** (*n* = 409)	**1** (*n* = 403)	**1** (*n* = 355)
Awakened sweating because of drinking	**1** (*n* = 358)	**1** (*n* = 310)	**2** (*n* = 337)	**0** (*n* = 414)	**0** (*n* = 405)	**0** (*n* = 358)
Experienced one or more withdrawal symptoms	**2** (*n* = 360)	**6** (*n* = 310)	**9** (*n* = 337)	**1** (*n* = 419)	**2** *(n* = 405)	**3** (*n* = 358)

a Each percentage was calculated based on the number of all drinkers in 1992 who did not report the symptom in 1984.

**Table 5:** Prevalence of Alcohol Withdrawal Symptoms in a Sample of White, Black, and Mexican-American Men Undergoing Alcoholism Treatment

	Ethnicity (%)		
	
Withdrawal Symptom	White (*n* = 258)	Black (*n* = 269)	Mexican-American (*n* = 221)
Shaking	70	29	56
Unable to sleep (i.e., insomnia)	82	54	65
Feeling anxious or depressed	86	63	68
Sweating	75	52	63
Rapid heart beat	52	26	46
Delirium tremens	29	11	24
Seeing/hearing things (i.e., hallucinations)	28	25	27
Nausea/vomiting	61	36	48
Weakness	71	56	61
Headaches	60	58	57
Fits/seizures	16	8	15
Drinking to stop withdrawal symptoms	75	46	61

NOTE: All *n’*s are unweighted.

**Table 6:** Prevalence of Alcohol Withdrawal Symptoms in a Sample of White and Mexican-American Men Undergoing Treatment After Being Convicted of Driving Under the Influence

	Ethnicity (%)	
	
Withdrawal Symptom	White (*n* = 229)	Mexican-American (*n* = 216)
Shaking	36	22
Unable to sleep (i.e., insomnia)	53	30
Feeling anxious or depressed	64	43
Sweating	36	32
Rapid heart beat	25	18
Delirium tremens	16	6
Seeing/hearing things (i.e., hallucinations)	13	6
Nausea/vomiting	34	25
Weakness	42	30
Headaches	59	62
Fits/seizures	14	3
Drinking to stop withdrawal symptoms	17	12
Withdrawal according to DSM-IV^a^ criteria	19	15

## Footnotes

1 For a definition of this and other technical terms used in this article, see glossary, p. 80.

## GLOSSARY

Confidence interval of an odds ratio: The 95-percent confidence interval is a range of values constructed around the estimate of an odds ratio obtained for a specific sample. The confidence interval represents the range of values that if the study was performed repeatedly, would capture the true value of the odds ratio 95 percent of the time.
Cross-sectional study: A study that compares the data of two or more groups of people (e.g., different ethnic groups) at one point in time.
Incidence: The number of new cases of a variable (e.g., withdrawal symptoms) occurring during a particular period of time.
Longitudinal study: A long-term study in which the same subjects are tested or interviewed two or more times during the study period.
Multistage area probability procedure: A sampling method that involves an initial sampling of groups of subjects, followed by the selection of subjects within each of those groups.
Odds ratio: A measure of association between two variables (e.g., alcohol consumption and presence of withdrawal symptoms).
Oversampling: The process of intentionally including a greater proportion of persons from a particular group (e.g., ethnic group) in a study than would correspond to their proportion in the general population.
Prevalence: The frequency with which a variable (e.g., withdrawal symptoms) occurs in a population at a certain point in time.
